# Results

**Published:** 1891-06-06

**Authors:** 


### The Hospital

A Weekly Journal of

### Science, Medicine, Nursing, and Philanthropy.

*For Hospitals, Asylums, Infirmaries, Dispensaries, Medical Practitioners, Students, Nurses, and the Charitable Public.*

Vol. X.—No. 245.] [June 6, 1891.

### Results.

Every shrewd man would pay by results if he could, and only by results. It is the prerogative of the divine distributor of justice to pay for honest intentions and strenuous strivings after the good and the beautiful. Triflers in philosophy love to discuss the question whether the pleasure is greater of pursuing a particular object, or of gaining it. Practical people, as a rule, have no doubt upon the subject; they love results; and no matter what be the pleasure of the hunt, they are always disappointed when they do not kill their fox.

The London hospitals spent last year a grand total of £606,258; about £12,000 a-week, or nearly £2,000 for every working day, which is equivalent to £200 an hour for ten hours daily. Bringing down the calculation still lower, we have an expenditure of almost £4 a minute, or considerably more than a shilling for every tick of the clock. This way of putting the thing appears rather like elaborate trifling, but it is eminently desirable that all sorts of minds should have a clear comprehension of the great and untiring efforts that are constantly being put forth in the cause of medical charity.

In the pursuit of all this money hospital officials spend a good deal of time, and not a little patience and ingenuity. We never yet heard, however, that they had more satisfaction in striving to get the money than in the actual handling of it when it has been secured. We are quite willing to believe that nineteen out of every twenty of them would gladly give up the hunt if a good fox, with a noble brush, would walk uninvited into their treasury. But few foxes seem to avail themselves, without persuasion, of this manifest privilege; on the contrary, packs of hounds have to be kept; masters, huntsmen, and whippers-in have to be called into requisition; and wide tracts of country have to be scoured from week to week in diligent pursuit of the indispensable cheques and bank notes.

Great results follow upon all these efforts; and the immediate results become, in turn, the par.nts of a large progeny of descendant results. The first manifest result of the activity of the London hospitals is the bringing in of £606,258 a year, or, as we have said, something over a shilling for every tick of the clock. The six hundred odd thousand pounds set in motion one million two hundred and thirty-six thousand human beings in the shape of patients. The gathered money represents the results for which hospital officials are more immediately anxious; the twelve hundred and thirty-six thousand patients, with their illnesses and accidents, their sorrows and sufferings, their cure and their consequent happiness, constitute the results which subscribers and all lovers of their kind concentrate their keenest interest upon.

A strange army these million and a-quarter of human beings constitute. They have begun their conflict with death; and the hospitals, with their physicians, surgeons, and nurses, have rushed into the thick of the fight against the universal and all-powerful enemy. Nearly eighty thousand of the million and a-quarter are in patients, which means that the enemy has succeeded in throwing this large proportion quite upon their backs; and some of them he has grasped so firmly by their vital parts that they will never rise again. A melancholy, not to say tragical march-past those eighty thousand would make if they could rise from their beds and march at all. The tragedy of human destiny could perhaps hardly be shown so clearly by any other eighty thousand people which we could bring together in this England of ours. The aged men with their palsied limbs and world-conquered looks, the aged women, too, with still more pathetic marks of resigned defeat upon them; the men in middle life with the tokens of disciplined submission to the inevitable in every line of their faces, and even in the cut of their beards; and the middle-aged women folk, also with expressions alternating between strenuous resolution and hopeless despair; and then the children, many of them sickly and deformed by sad inheritance; others maimed by accident, and still others tossed and tormented by the anguished pains and burning fevers which bad sanitation produces. A terrible army: forcing us sometimes to ask Where is God? What is the use of human government? Where is the practical helpfulness that science never ceases to vaunt? This is the permanent spectacle that London puts on her public stage; and puts on show afresh with every advancing year. And if such be the spectacle that London cannot hide, what would be the intolerable nature of the sight that all the sick and ill of the civilised and uncivilised world would reveal if they could be brought together in one terrible procession that would hardly know an end?

There is no indulgence in exaggerated rhetoric here. If all the sick poor of the world, with their deformities, their maimed limbs, their feeble, prostrate, and anguished forms, and the marks of vicious and pauperised inheritance upon them, could be brought together in a vast living tableau, the sight would dim the eyes and rend the hearts of all men. What would every one of us straightway wish to do? An overpowering influence would seize upon us to gather them into a protecting fold; we should want to send gentle and capable nursing women amongst them; and next we should call for the kind physician and the skilled surgeon; and we should feel a relief and a contentment of heart indescribable when we had seen the last of them enter within the door of the protecting fold, and lie down on his bed where human kindness, science, and practised skill would give him their divine ministrations.

In England hospitals have brought about this altogether blessed result for us. Is such a result in London worth the £600,000 a year we pay for it? Let the sick and the suffering themselves answer the question! Listen to the chorus of mingled voices, the quavering notes of the aged, the deep-toned utterances of the men in middle life, and the sweet treble of the children. Nothing which the sick can say or do can approach the expression of the gratitude they feel. Great is the result to the sick of all the money that is collected and all the knowledge and skill that are used on their behalf. But is there not an even greater result to the prosperous and the healthy? Could we endure the thought of eighty thousand in-patients in London alone, so seriously ill as to be quite helpless, overwhelmed in poverty, and left uncared for to die or live in their small and squalid homes? The thing would be intolerable. No man with an English heart, or with a human heart of any kind, could bear the thought without pain and shame; still less could our gentle mothers and wives and sisters enjoy their innocent home and social pleasures if the sick and the injured and the poor were left without help. Civilization and religion have done a great thing for us in that they have trained our hearts to such tenderness and justice that we cannot see our brethren starve without wishing to share our own food with them, or witness their bodily suffering and anguish without striving to bear our part, and to do all that may lie within our power to alleviate their sorrows and pains.

Last week we published our special Hospital Sunday number. But Hospital Sunday is still two days off when this number is issued. There is yet time for all of us to give a fresh thought, a deeper and wider thought, perhaps, to the great army of sick and suffering who are now within the enfolding protection of the hospitals, or who will need that gracious protection during the twelve months immediately before us. The fresh thought, let us hope, will be generous and tender. If it were only to cause each reader to give twice as much as he intended to give to the Hospital Sunday collection, there would be to that extent a double result of the most beneficent of all beneficent activities. Who among us all will be persuaded to give a double blessing to his poor fellow citizens, whose fortune it may be to be carried sick or injured to the hospital before the end of the philanthropic year upon which we are now entering? It is not given to men and women in this world to do any more truly divine work than caring for and helping the helpless sick. We make no appeal to strained sentiment or to artificially-produced enthusiasm. The sick are with us, and they will continue to be with us. Their own helplessness is their most eloquent plea. If England may thank God for anything, she may thank Him most of all that He has placed her at the head of all nations and peoples in the fulness of her regard for those who are in any sort of need, sickness, or distress. The courage that carries our arms and arts to the remotest regions of the earth is the twin sister of the tenderness that makes every form of human helplessness our peculiar and honourable care.

